# Prevalence of long gun use in Maryland firearm suicides

**DOI:** 10.1186/s40621-019-0230-y

**Published:** 2020-02-03

**Authors:** Paul S. Nestadt, Kevin MacKrell, Alexander D. McCourt, David R. Fowler, Cassandra K. Crifasi

**Affiliations:** 1 Department of Psychiatry and Behavioral Sciences, Johns Hopkins University School of Medicine, Baltimore, USA; 2 Department of Mental Health, Johns Hopkins Bloomberg School of Public Health, Baltimore, USA; 3 Johns Hopkins University School of Medicine, Baltimore, USA; 4 Department of Health Policy and Management, Johns Hopkins Bloomberg School of Public Health, Baltimore, USA; 5 Office of the Chief Medical Examiner of Maryland, Baltimore, USA

**Keywords:** Firearms, Suicide, Long guns, Policy, Rurality, Epidemiology

## Abstract

**Background:**

Firearms account for the majority of US suicides, largely due to lethality and accessibility. Under Federal and Maryland law, long guns are less regulated than handguns which is a concern for increased suicide risk. This study uses Maryland data to ascertain the impact of long guns on suicides in the state. We hypothesize that the prevalence of long gun use among firearm suicides will be increased in rural and young populations.

**Methods:**

This is a cross sectional study using police and medical examiner narratives to identify firearm type involved in all 3931 Maryland gun suicides from 2003 to 2018. Proportions of firearm suicides utilizing long guns were calculated. Urban-rural differences were determined using the National Center for Health Statistics’ classification system. Logistic regression was used to calculate odds ratios of long gun to handgun suicides across the urban-rural spectrum, controlling for decedent demographics.

**Results:**

From 2003 to 2018, 28.4% of Maryland gun suicides used long guns. The proportion of long guns used was highest in the most rural counties, where 51.6% of firearm suicides were by long gun, compared to 16.8% in the most urban counties. Long guns were disproportionately used by the young. For decedents 18 or younger, 44.6% used long guns, compared to 20.2% in those 65 or older. Compared to the most urban counties, firearm suicide decedents in the most rural counties were 3.74x more likely to use long guns (OR = 3.74; 95% CI 2.19, 6.40; *p* < .001) after adjusting for demographics, intoxication, and hunting season.

**Conclusions:**

Long guns are used in a large proportion of Maryland firearm suicides, particularly in rural areas and disproportionately in youth suicides. Long guns must be considered as part of access to lethal means or policy strategies in efforts to reduce the burden of firearm suicide.

## Background

In the United States in 2017, there were more than 47,000 suicides (Center for Disease Control and Prevention 2019). While firearms are used in a small proportion of overall attempts, they account for more than half of fatal suicides, due in large part to a case fatality rate of more than 80% (Spicer and Miller 2000; Vyrostek et al. 2004; Conner et al. 2019). A significant proportion of suicide attempts are made impulsively, with the decision to act occurring within an hour of the attempt in 71% of cases, and within less than 5 min in 24% of cases (Deisenhammer et al. 2009; Simon et al. 2001). Given this short time between decision and action, it is unsurprising that the method used reflects method availability (Eddleston et al. 2006; Peterson et al. 1985), and if that method has a high lethality it will by definition result in higher mortality. Coupled with the fact that attempt survivors rarely die in subsequent attempts (O’Donnell et al. 1994; Seiden 1978), and most suicide decedents have no history of a past attempt (Cavanagh et al. 2003), access to highly lethal means has been recognized as one of the most important contributors to high rates of completed suicide.

Multiple studies have established a consistent association between household firearm ownership and increased suicide rates (Anglemyer et al. 2014; Butterworth et al. 2018; Shenassa et al. 2004; Kposowa et al. 2016; Dahlberg et al. 2004; Wiebe 2003). State laws that have reduced access by high risk individuals such as mandating background checks through purchaser licensing, waiting periods, and child access protection laws have all been found to reduce suicide rates individually and measured together (Anestis and Anestis 2015; Crifasi et al. 2015; Kaufman et al. 2018; Loftin et al. 1991; Webster et al. 2004).

Almost 80% of firearm homicides and 90% of nonfatal firearm injuries are committed with a handgun (Planty and Truman 2013), and this may guide the stricter regulations on handguns in comparison to long guns both federally and across states (Cook et al. 2011). While the majority of firearm deaths are self-inflicted, there has been limited research on the type of firearm used in suicides. A recent study using the Centers for Disease Control and Prevention’s National Violent Death Reporting System (NVDRS) data for 13 states found that 27% of firearm suicides from 2005 to 2015 utilized a long gun, with greater proportions found in rural populations and young decedents (Centers for disease control and preventio 2019; Hanlon et al. 2019). Given the heterogeneity of states, there is a need for similar state-level investigations to describe this burden and assist in determining the need for interventions such as policy, education, screening, and safe storage initiatives aimed at reducing firearm suicide. This study examines the role of long guns in firearm mortality, particularly suicides, in a state which is currently grappling with the question of long gun regulation.

In Maryland, long gun ownership is less regulated than handgun ownership, especially in regard to age limits. In Maryland, it is prohibited for any person (licensed dealer or private individual) to sell or transfer a handgun to anyone under 21 and anyone under 21 is prohibited from possessing a handgun, yet there is no minimum age for possessing a rifle or shotgun (Md. Code Ann., Pub Safety § 5–134 n.d.). State regulations requiring waiting periods, permits to purchase and carry, and licensing of owners for handguns do not apply to long guns (Md. Code Ann., Pub Safety § 5-101(r)). These long gun exceptions in Maryland may increase the accessibility of long guns, especially to young people.

The increased accessibility of long guns has the potential to disproportionately impact rural areas where suicide rates are consistently higher than in urban counties, and the gap has increased dramatically over the past 10 years (Kegler et al. 2017). A recent study comparing urban to rural suicides in Maryland found that the increased rural rates were driven by firearm use (Nestadt et al. 2017). In general, rural decedents are more likely to use a firearm compared to urban decedents (Branas et al. 2004; Searles et al. 2014), and in a survey of rural firearm owners approximately 90% of households contained a long gun (Nordstrom et al. 2001). Data on long gun suicides may help inform public health initiatives and educational outreach to address the disproportionate impact on rural and young populations.

Federal restrictions on the collection and analysis of firearm data has resulted in a lack of detailed data on firearm ownership by region and demographics, as well as on the models of firearm used in suicide (Rostron 2018). However, individual police reports and medical examiner death scene investigations do generally document firearm types and can be accessed at the state and local level.

The current research examines the role of rifle and shotgun suicides in Maryland, with a focus on rurality and hunting season as potentially affecting risk. We hypothesize that long guns play a significant role in Maryland’s firearm deaths and in particular suicides. We further hypothesize that rurality, as well as other demographic and clinical factors, will affect long gun use among firearm suicide decedents. Secondarily, we will test an association between hunting season and long gun use for suicide and investigate whether these associations differ between the two types of long guns: rifles and shotguns.

## Methods

### Population and procedures

The Office of the Chief Medical Examiner of Maryland (OCME) investigates all violent, sudden, suspicious, or unexpected deaths, as well as any death without a physician in attendance, and deaths in a penal institution in the state of Maryland. Maryland established the country’s first state-wide protocolized medical examiner system, which guarantees consistency in investigation throughout the state, unlike other data sets which use a heterogeneous mix of coroner and medical examiner systems with different standards for classification of suicide, varying levels of investigative depth, and inconsistent reporting standards. There have been no significant changes in medical examiner investigative or recording procedures since 2002 and OCME leadership has been consistent during the study period.

Our study is a cross sectional study using a complete listing for all 3994 non-homicide firearm deaths in Maryland from 2003 to 2018, including 3931 suicides, 29 unintentional deaths, and 34 deaths of undetermined manner. As described in previous publications (Nestadt et al. 2017), a board-certified psychiatrist (PSN) reviewed 5% of these cases via police and OCME reports, and then interviewed the chief medical examiner to confirm agreement with the OCME definitions of suicide. From the OCME records, we extracted age, sex, race/ethnicity (as defined by the OCME), county of residence, method of suicide, toxicology, and both OCME and police reports on the deaths.

### Measures

#### Firearm type

Using police narratives, we coded the type of firearm used for each firearm death in the sample into either handgun or long gun (rifle or shotgun), based on the police report’s documenting of the weapon as handgun, shotgun, or rifle or in some cases by the specific model of weapon, which would be categorized accordingly. Among the 3994 deaths described in the dataset, there were 46 incidents noted for disagreements between police and medical investigation reports as to whether the long gun used was specifically a rifle or a shotgun. In these cases, which represented 1% of the total, PSN and KM performed manual reviews of the case reports to clarify the weapon type. The added detail in the narratives of the report usually made it clear which reporter was correct based on a specific model listed, which could be researched in the firearm maker’s catalogue and classified as rifle or shotgun by manufacturer description. In all cases, they agreed on the ultimate determination. Of these, 22 were reclassified as rifles and 22 shotguns, with 1 case where the weapon was found to be a handgun and 1 case where the technical cause of death was in fact hanging.

#### Demographics

We collected the sex, age, and race for each decedent from the OCME reports. To examine urban-rural differences, we used the county of residence for each decedent, categorized using the National Center for Health Statistics (NCHS) Urban-Rural Classification Scheme for Counties. This scheme designates counties into 6 different classifications on the basis of population size and adjacency to metropolitan areas, with the lowest category number indicating the highest degree of urbanicity. The first 4 categories – Large Central Metro, Large Fringe Metro, Medium Metro, and Small Metro – fall under the metropolitan level and the last two categories – Micropolitan and Noncore – are considered nonmetropolitan. For directly comparing urban versus rural differences in firearm suicides, we collapsed the first 4 categories into one single urban category and the remaining two categories into one rural category, in accordance with their characterization in the NCHS.

#### Intoxication

Blood alcohol content (BAC) was recorded by the OCME from peripheral blood when the body was recovered quickly and estimated from aqueous humor when peripheral blood was not available before significant body decomposition. A decedent was considered to have been intoxicated on alcohol when the BAC collected in this manner was greater than .08%. Sex was determined by the medical examiner based on the physical exam at autopsy. Age was categorized into 5 bins, representative of youth (< 18 years), young adult (18–24), adult (25–44), middle age (45–64), and older (65 and older), to match categories used in previous studies of firearm type in suicide (Hanlon et al. 2019).

#### Hunting season

Hunting season was operationalized based on the Maryland Deer Hunting report (Eyler and Timko 2018), which tabulates that the majority of the firearm involved deer harvest was reported to occur in the 2 week season beginning on the Saturday after Thanksgiving, which moves year to year. Therefore, we operationalized hunting season throughout the study period as a binary variable positive for deaths in annual weeks 49 and 50, which approximates this time period over the years of the study.

We did not use the availability of mental health providers as a covariate in our analyses because a previous study using data from the OCME during the same time period found that there were no significant differences in mental health care provider availability across urban–rural counties of Maryland (Nestadt et al. 2017).

#### Analytic plan

Proportions of firearm suicides using long guns were calculated separately for each decedent characteristic. Odds ratios were calculated for each characteristic using single variable logistic regression. Proportions of long gun suicides for urban and rural demographics were computed using the NCHS categories explained above and age was categorized into the previously described five age ranges (< 18, 18–24, 25–44, 45–64, and > 65), with logistic regressions performed using the most urban and youngest categories respectively as references.

The proportion of long gun suicides among all firearm suicides were then calculated for each age range, stratifying by rurality, with NCHS categories 1–4 considered urban and 5–6 considered rural. In order to assess for an interaction between rural status and age category in predicting long gun use, an interaction term was created and tested in a logistic regression model.

Proportions of firearm suicide decedents who had used rifles or shotguns were separately tabulated, with χ^2^ tests performed to establish differences by decedent characteristics within each firearm type group.

To further evaluate the hypothesis that rurality may predict long gun use, a series of logistic regression models were tested, first adding adjustments for sex, race, and age (Model 2) because these are known to influence suicide rate and have been hypothesized to also correlate with long gun ownership. A third model was then tested with adjustments for alcohol intoxication and hunting season death (Model 3), as these were found to be associated with suicide rates in unadjusted analyses. We conducted all analyses with Stata version 16.0 (StataCorp LP, College Station, TX).

## Results

Our data query returned 3994 charts. The vast majority of firearm suicide decedents (82%) were non-Hispanic whites, with the remaining 18% distributed among several race and ethnicity categories, and so for the purposes of this study, decedents were categorized as either white or non-white. Race and/or age data was missing from only 10 cases. As the combined number of decedents missing either of these variables added up to less than 1% of the cohort, we continued analysis after listwise deletion of cases with these missing data. Toxicology was unavailable for 253 (6.4%) of the suicide decedents, usually due to the body not being recovered in time for accurate toxicology to be performed, and so these cases were dropped from any analysis which included BAC. In 101 cases (2.5%) the decedent county of residence was not in Maryland, and in 127 cases (3.2%) the county of residence was unknown. These cases were dropped from any analysis that took rurality into account.

Of all 3994 non-homicide firearm deaths studied, 1134 (28.4%) were caused by long guns. Among these, there were 29 unintentional firearm deaths, 6 (20.7%) were due to long guns including 3 shotguns and 3 rifles. There were 34 firearm deaths of undetermined manner, 12 (35.3%) were by long gun including 10 shotguns and 2 rifles. The remaining non-homicide firearm deaths were suicides. Of the 3931 firearm suicides 1116 (28.4%) used long guns. Of these, 786 (70.4%) were via shotgun and the remaining 330 (29.6%) via rifle (Additional file 1: Figure S1).

Although the proportion of firearm deaths by long gun were calculated for each manner of death, only suicide deaths included a sufficiently large sample for the remainder of the analysis described below.

A breakdown of type of firearm used by decedent characteristics is presented in Table 1. Among these firearm suicides, long gun use was more prevalent in men than women (OR = 2.4; 95% CI = 1.81, 3.16; *p* < .001) and more common in whites than non-whites (OR = 2.3; 95% CI = 1.83, 2.79; *p* < .001).

**Table 1:** Characteristics of Maryland Firearm Suicides 2003–2018, with Unadjusted Odds Ratios

Variables		Handgun *n* = 2815 (row %)	Long Gun *n* = 1116 (row %)	Odds Ratio	95% CI	*p*
Sex*						
Female		347 (84.8%)	62 (15.2%)	*ref*	–	–
Male		2468 (70.1%)	1054 (29.9%)	2.39	1.81–3.16	< .001
Race*						
Non-White		589 (83.4%)	117 (16.6%)	*ref*	–	–
White		2226 (69.0%)	999 (30.1%)	2.26	1.83–2.79	< .001
Age (years)*						
< 18		46 (55.4%)	37 (44.6%)	*ref*	–	–
18–24		222 (62.9%)	131 (37.1%)	.73	.45–1.19	.209
25–44		749 (70.9%)	308 (29.1%)	.51	.33–.80	.004
45–64		1002 (69.5%)	439 (30.5%)	.54	.35–.85	.008
65+		796 (79.8%)	201 (20.2%)	.31	.20–.50	< .001
NCHS Rurality*						
Urban (1)		233 (83.2%)	47 (16.8%)	*ref*	–	–
2		1986 (72.6%)	750 (27.4%)	1.87	1.35–2.59	< .001
3		206 (65.6%)	108 (34.4%	2.60	1.76–3.84	< .001
4		130 (64.4%)	72 (35.6%)	2.75	1.79–4.20	< .001
5		46 (56.1%)	36 (43.9%)	3.88	2.27–6.64	< .001
Rural (6)		46 (48.4%)	49 (51.6%)	5.28	3.17–8.79	< .001
Alcohol (> .08%)*		578 (68.0%)	272 (32.0%)	1.21	1.03–1.43	.022
Season						
Non-Hunting		2720 (71.7%)	1074 (28.3%)	*ref*	–	–
Hunting Season		95 (69.3%)	1116 (28.4%)	1.12	.77–1.62	.549

*NCHS Rurality* National Center for Health Statistics Urban-Rural Classification Scheme for Counties. Hunting Season includes annual weeks 49–50

*N*’s range from 3675 to 3931 due to occasional missing data

*Category χ^2^ test *p* < .001

Increasing rurality as well as younger age were both strongly associated with increasing proportions of long gun use, in a graded fashion. Compared to the most urban decedents, the most rural decedents were more than 5x as likely to have used a long gun (OR = 5.3; 95% CI = 3.17, 8.79; *p* < .001), with each level of rurality associated with increasing likelihood. Significant age differences were also found, with decedents younger than 18 years demonstrating the highest proportion (44.6%) of long gun use, compared to much lower rates (20.2%) in those 65 or older (OR = .31; 95% CI = .20, .50; p < .001). During deer hunting season in Maryland, firearm suicide decedents were no more likely to have used a long gun.

Rifles and shotguns were then evaluated separately, and hunting season was found to correlate with a higher proportion of rifle use among firearm suicides, χ^2^_(1, *N* = 3931)_ = 4.15, *p* = .042). There was a nonsignificant decrease in shotgun use for suicide during hunting season. The established increased use of long guns in whites, males, and youth was found to be more pronounced in rifle than shotgun use, as shown in Table 2. However, the primary finding of increasing long gun use with increasing rurality was much more pronounced in shotguns, though still strong in rifles. Shotgun users were also significantly less likely to be intoxicated at the time of their suicide.

**Table 2:** Characteristics of Rifle and Shotgun Use Among Firearm Suicides

Variables	Total n = 3931	Rifle *n* = 330 (%)	χ^2^	*p*	Shotgun *n* = 786 (%)	χ^2^	*p*
Sex							
Female	409	14 (3.4%)	14.7	< .001**	48 (11.7%)	19.5	< .001**
Male	3522	316 (9.0%)			738 (21.0%)		
Race							
Non-White	706	29 (4.1%)	20.6	< .001**	88 (12.5%)	30.5	< .001**
White	3225	301 (9.33%)			698 (21.6%)		
Age (years)							
< 18	83	18 (21.7%)	21.3	< .001**	19 (22.9%)	51.5	< .001**
18–24	353	30 (8.5%)			101 (28.6%)		
25–44	1057	92 (8.7%)			216 (20.4%)		
45–64	1441	119 (8.3%)			320 (22.2%)		
65+	997	71 (7.1%)			130 (13.0%)		
NCHS Rurality							
Urban (1)	280	12 (4.3%)	13.1	.022*	35 (12.5%)	47.3	< .001**
2	2736	222 (8.1%)			528 (19.3%)		
3	314	35 (11.2%)			73 (23.3%)		
4	202	22 (10.9%)			50 (24.8%)		
5	82	9 (11.0%)			27 (32.9%)		
Rural (6)	95	11 (12.6%)			38 (40.0%)		
Alcohol (BAC)							
< 0.08%	2825	239 (8.5%)	.19	.661	550 (19.5%)	5.2	.023*
> 0.08%	850	76 (8.9%)			196 (23.06%)		
Season							
Non-Hunting	3794	312 (8.2%)	4.15	.042*	762 (20.1%)	.5	.461
Hunting Season	137	18 (13.1%)			24 (17.5%)		

*NCHS Rurality* National Center for Health Statistics Urban-Rural Classification Scheme for Counties. Hunting Season includes annual weeks 49–50. *BAC* Blood Alcohol Content

*N*’s range from 3675 to 3931 due to occasional missing data

* *p* < .05 ** *p* < .001

**Fig. 1:**
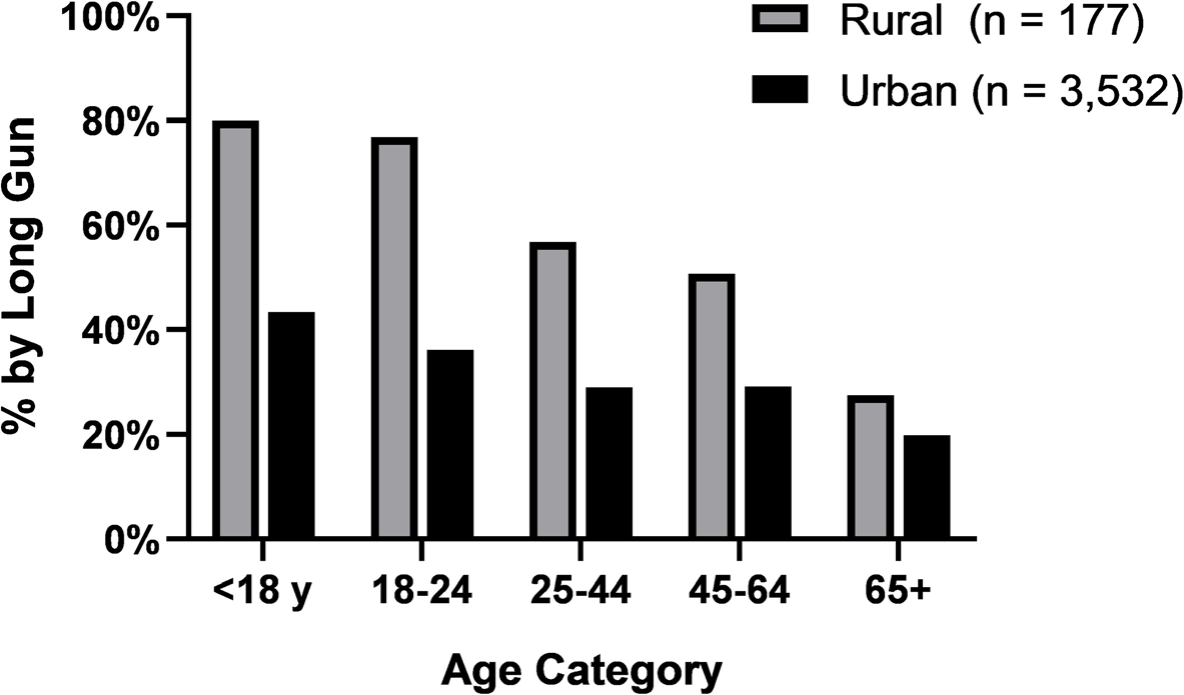
Proportion of Firearm Suicides Involving Long Guns, by Age and Rurality. NCHS categories 1–4 are considered urban and categories 5–6 are rural

The proportion of long gun suicides by age group stratified by urban and rural categories is shown in Fig. 1. Within both categories of rurality, the proportion of long gun suicides decreased with age, while remaining disproportionately rural in each age group. Among rural firearm suicide decedents under 18, 4 out of 5 decedents (80%) had used a long gun, while only 43% of their urban counterparts had done so. This trend was repeated within every age category, though the differences became less stark with increasing age. In logistic regression, an interaction between rural status and age category was found to be significant (OR = .68; 95% CI = .49, .96; *p* = .030), indicating that the increasing proportions of long gun use with younger age were more dramatic in rural populations than urban.

The relationship between rurality and long gun use was further tested using a series of sequentially adjusted regression models, displayed in Table 3. Model 1 illustrates the relationship between the categories of rurality and the use of a long gun to be quite robust in unadjusted analysis, with the most rural counties demonstrating 5.3 times the proportion of long gun suicides compared to the most urban counties, as well as a linear increase in long gun use across the urban-rural spectrum. In Model 2, adjustments were made for age, sex and race which had demonstrated significance in distinguishing long gun use from handgun use in firearm suicides. Adjusting for age, sex, and race did not negate the correlation with rurality. A final model, Model 3, added adjustmentsfor alcohol intoxication and hunting season, as these had been hypothesized to contribute to the availability and hazard for long gun use in suicide, though did not demonstrate the larger associations with long gun use for suicide seen in univariate analysis of age, sex and race. Although this adjustment diminished the finding, with the odds ratio comparing the most rural to most urban decedents dropping to 3.7, the finding remained significant across all NCHS categories in this and all other models.

**Table 3:** Stepwise Logistic Regression Analysis with Odds for Predictors of Long Gun Use Among Firearm Suicides

Variable	Model 1			Model 2			Model 3		
	OR	95% CI	*p*	OR	95% CI	*p*	OR	95% CI	*p*
NCHS Rurality									
Large metro (1)	*ref*	–	–	*ref*	–	–	*ref*	–	–
Large fringe metro (2)	1.87**	1.35–2.59	< .001	1.61*	1.15–2.25	.005	1.49*	1.06–2.09	.021
Medium metro (3)	2.60**	1.76–3.84	< .001	2.20**	1.47–3.30	< .001	2.03**	1.34–3.07	< .001
Small metro (4)	2.75**	1.79–4.20	< .001	2.00*	1.29–3.12	.002	1.89*	1.21–2.95	.005
Micropolitan (5)	3.88**	2.27–6.64	< .001	3.27**	1.88–5.70	< .001	3.00**	1.70–5.30	< .001
Noncore (6)	5.28**	3.17–8.79	< .001	4.22**	2.49–7.15	< .001	3.74**	2.19–6.40	< .001
Male				2.68**	2.00–3.59	< .001	2.93**	2.16–3.97	< .001
White				2.54**	2.01–3.21	< .001	2.56**	2.02–3.26	< .001
Age (y)				.98**	.98–.99	< .001	.98**	.98–.99	< .001
Alcohol > .08%							.98	.82–1.17	.834
Hunting Season							1.18	.79–1.77	.423

*NCHS Rurality* National Center for Health Statistics Urban-Rural Classification Scheme for Counties. Hunting Season includes annual weeks 49–50. *N*’s range from 3675 to 3931 due to occasional missing data

*CI* confidence interval, *OR* odds ratio calculated by multivariate logistic regression

* *p* < .05 ** *p* < .001

## Discussion

Our findings reveal that in Marylandlong guns are responsible for a substantial proportion of non-homicide firearm deaths. Among firearm related deaths by unintentional or undetermined manner, which are much less common than suicides, 28.6% were caused by long guns, primarily shotguns. Although the sample of non-suicide firearm death was limited to 63 cases, to our knowledge this was the first study to investigate firearm type in these deaths.

We found that 28.4% of firearm suicides used long guns, with 70.4% of these being shotguns. This proportion of long gun suicides was consistent with Hanlon et al.’s (2019) previous study using national data across all age groups which found that 27% of firearm suicides used long guns. These findings were also similar to a small study of Sacramento County, which found that 31% of firearm suicides were by long gun, though they noted that rifles were twice as commonly used as shotguns in that sample. This may reflect increased prevalence of rifles over shotguns in that region, as that study included survey data from the Pacific census division reporting 40% of self-reported firearms were rifles and only 26% shotguns (Wintemute et al. 1998). National surveys conducted by the Pew Research Center also found that while 62% of firearm owners reported that they owned a rifle, only 54% owned a shotgun (Parker et al. 2017). Maryland’s proportion of rifle and shotgun ownership is not known, but future studies may develop local surveys for this purpose and address this unresolved question while exploring alternate reasons for the increased proportions of shotgun suicides.

Consistent with an earlier national study (Hanlon et al. 2019), the present study found that the use of a long gun for suicide was more common in whites and men, and that long guns demonstrated increasing use with increasing rurality and decreasing age. We also found that for rifles, which are the most common weapon used for hunting, the proportion of suicides increased dramatically during deer hunting season. This did not hold true for other firearm types. Previous studies have found that alcohol intoxication is associated with the choice of more violent and lethal suicide methods—hanging and firearm use as compared to overdose or other poisonings—and that this association is strongest in younger decedents (Conner et al. 2014). We considered that this may also apply to the use of long guns, which may be seen as more lethal than smaller weapons, and did find this to be the case with intoxicated decedents choosing to use a long gun 21% more often than sober decedents.

Research has consistently shown that the accessibility of a particular method is the primary determinant of its use in suicide (Anglemyer et al. 2014; Butterworth et al. 2018; Kposowa et al. 2016; Spicer and Miller 2000; Vyrostek et al. 2004). While the relative ownership of long guns to handguns in Maryland is not known, we do know that the most common reported purpose for having a long gun is hunting (Wolfson et al. 2018). Rural residents are more likely to hunt, and over 90% of hunters are white and/or male (U.S. Fish and Wildlife Service. 2018; Wilson et al. 2012). These demographics may explain the disproportionate long gun use in rural areas. Interestingly, the increase in long gun use over successively more rural counties remained strongly significant even after adjusting for demographics, alcohol intoxication, and hunting season deaths.

In addition to rurality, the use of a long gun increased with decreasing age, with 45% of decedents under 18 using long guns compared to 28% of decedents 18 and over. As shown in Table 3, this correlation with age did not dissipate when controlling for other characteristics. Suicides are often impulsive, especially in the young (Deisenhammer et al. 2009; Simon et al. 2001), and younger people have been shown to have greater increases in suicide rates when exposed to a firearm in the home than those 18 and over (Miller et al. 2015). The increased odds of suicide by long gun may reflect long guns being harder to hide and more expensive to lock away from children. As shown in Fig. 1, 80% of rural youth firearm suicides were by long gun and there is a steeper age-based correlation in rural counties, where the young may be more likely to have access to weapons for hunting purposes. The significance of the interaction between rural status and age group may reflect the popularity of hunting using rifles and shotguns among young people, more commonly in rural than urban areas.

Another potential explanation for the use of long guns by younger decedents may be the lack of legal restrictions on their owning these weapons or purchasing them in private sales. Aside from those who may already have a firearm in the home, the ability to immediately purchase long guns at a young age, without background check or waiting period, makes them more accessible to that demographic than handguns.

Future studies should investigate how changes in regulatory policies, such as child access protection laws, licensing, and age limits which bring long gun requirements in line with those of handguns may help to reduce youth suicide rates. These findings provide a detailed breakdown of the demographic-specific dangers of rifles and shotguns, but future studies might look into the ways in which long guns were accessed leading to a suicide, including whether they tend to be purchased soon before use, or if they are long owned or even family heirlooms. Patterns of safe storage should be investigated, as long guns may lend themselves to different forms of storage or be more likely to be on display, and hence accessible to those at risk.

While firearm suicide may be best seen through a public health lens with policy-level solutions (McLean et al. 2019), this work also provides valuable insights for the clinician focused on suicide prevention on a patient-by-patient basis. While universal screening for suicidal ideation may have a limited impact on completed suicides (Nestadt et al. 2018), discussing with patients about their access to firearms and counseling them on reducing access and safe storage of firearms is an effective and evidence based practice (Yip et al. 2012), included in national guidelines (U.S. Department of Health and Human Services 2012), though unfortunately underutilized (Betz et al. 2018; Roszko et al. 2016). In rural communities, where hunting is common and exposure more frequent, long guns may not be considered especially dangerous compared to handguns and so may not be considered when clinicians ask about firearms in the home. In much the same way that clinicians must specify over the counter medicines and supplements when taking a medication history, long gun access should be queried directly in a complete assessment. This is particularly the case in rural populations and for the highest risk patients, such as white men. Young patients and their parents should be reminded of the importance of safe storage and the removal of firearms during a crisis. New tools may be utilized to encourage safe storage at the point of care, taking firearm type into account (Betz et al. 2019).

Hunters may be at increased risk and interventions which focus on this population may include safe storage pamphlets and suicide crisis resource brochures at hunting stores and firing ranges, as well as in materials distributed in hunting forums and media. This underscores the importance of local partnerships with groups like the Gun Shop Project, which leverages the concern of firearm owners and others in the firearm industry to disseminate information on firearm safety and suicide risk directly to retailers and hunting supply shops (Henn et al. 2019). These firearm-owner led outreach and education groups have been effective in reaching rural gun owners and reducing firearm suicides, and equivalents have already gained traction in Maryland (Barber et al. 2017). While legislatively reducing access to firearms is less feasible, this data may be helpful in the ongoing efforts to promote safe storage practices, which may be spearheaded by hunting supply retailers themselves (Pierpoint et al. 2019; Tung et al. 2019). When trusted retailers are made aware of the increased use of long guns in suicides in the rural areas they serve, as well as the increase in rifle use for suicide during hunting season, they may be more likely to pass on these warnings to at risk customers and families.

### Strengths and limitations

There were several strengths to this study that support the validity and importance of the findings. The use of primary data from the state medical examiner for the purpose of detailing and confirming firearm type is unprecedented, as far as we are aware. These data are more current, more complete, and more consistently collected than national data sources derived from a mixture of coroner and medical examiner systems (Blair et al. 2016; Hanzlick 2006). A single state focus allowed a consistent hunting season variable to be defined and investigated, as hunting season does vary from region to region in the US, depending in part on the popular game. Maryland is an opportune study setting, not only because of the completeness of data afforded by a statewide medical examiner system, but for its wide range of rurality across counties ranging from the very urban Baltimore to swathes of rural western Maryland and the Eastern shore.

There are several limitations to this study. Choosing to focus on one state came with a cost to sample size. However, despite the lower power, the results maintained significance even when adjusted for important covariates. Another limitation of a single state study may be limited generalizability. Results found in Maryland may not apply to other states, such as those with extremes of rurality or different racial makeups. Finally, although no legislation specifically impacting long guns passed during the study period, changes in legislation during the study period were not controlled for, and may have affected the death by firearm rates.

## Conclusions

These findings draw attention to the role of long guns in contributing to firearm suicides, which are disproportionately used in rural and youth suicides. These findings highlight the need for more research to determine why youth are more likely to use long guns, and whether this is due to increased access due to lower age limits and more cultural acceptance of child possession of long guns. This research also has important implications for the need to educate communities, public health practitioners, law enforcement, and other key stakeholders about the important role firearm access plays in suicide prevention, particularly among rural youth. Addressing access to lethal means and increasing targeted screening should also be part of a concerted effort to prevent death and injury. Long guns are an important factor that should not be ignored by policy makers or clinicians as they work to reduce the burden of firearm death in the US.

## Supplementary information

**Additional file 1: Figure S1.** (A) The proportion of non-homicide firearm deaths stratified by firearm type and the manner of death (unintentional, undetermined, or suicide). (B) Long gun suicides are further stratified in a pie chart to show the breakdown between shotguns and rifles used in suicide.

## Data Availability

The data that support the findings of this study are available from the Office of the Chief Medical Examiner of Maryland but restrictions apply to the availability of these data, which were used under license for the current study, and so are not publicly available. Data are however available from the authors upon reasonable request and with permission of Office of the Chief Medical Examiner of Maryland.

## Abbreviations

BAC: Blood alcohol content
CDC: Centers for Disease Control and Prevention
NCHS: National Center for Health Statistics
NVDRS: National Violent Death Reporting System
OCME: Office of the Chief Medical Examiner of Maryland
