# Supplementary material for: Prevalence of long gun use in Maryland firearm suicides
Source: Inj Epidemiol. 2020 Feb 3;7:4. doi: 10.1186/s40621-019-0230-y (PMC6996182; doi:10.1186/s40621-019-0230-y)
Supplement: Supplementary file 1 — Additional file 1: Figure S1. (A) The proportion of non-homicide firearm deaths stratified by firearm type and the manner of death (unintentional, undetermined, or suicide). (B) Long gun suicides are further stratified in a pie chart to show the breakdown between shotguns and rifles used in suicide. [file 40621_2019_230_MOESM1_ESM.docx]

B

*Figure S1:* (1A) The proportion of non-homicide firearm deaths stratified by firearm type and the manner of death (unintentional, undetermined, or suicide). (1B) Long gun suicides are further stratified in a pie chart to show the breakdown between shotguns and rifles used in suicide.
